# StageTip: a little giant unveiling the potential of mass spectrometry-based proteomics

**DOI:** 10.1007/s44211-025-00749-1

**Published:** 2025-03-26

**Authors:** Eisuke Kanao, Yasushi Ishihama

**Affiliations:** 1 Graduate School of Pharmaceutical Sciences, Kyoto University, Kyoto, 606-8501 Japan; 2 Laboratory of Proteomics for Drug Discovery, National Institute of Biomedical Innovation, Health and Nutrition, Ibaraki, Osaka 567-0085 Japan

**Keywords:** Proteomics, StageTip, Sample preparation, LC/MS/MS

## Abstract

This review highlights the growing impact of StageTips (Stop and Go Extraction Tips), a pipette tip-based LC column in MS-based proteomics. By packing standard pipette tips with reversed-phase, ion-exchange, or metal oxide materials, StageTips enable efficient peptide desalting, fractionation, selective enrichment, and in-tip reactions with minimal sample loss. Recent improvements, including new resin designs and integrated workflows, have further expanded the applications to phosphoproteomics, protein terminomics, and single-cell proteomics. With their simplicity, high reproducibility, and low cost, StageTips offer a versatile platform that can be seamlessly integrated into automated pipelines, increasing the throughput and the depth of proteome analysis. As materials and protocols continue to evolve, StageTips will continue to develop as an essential keystone for robust sample preparation in next-generation proteomics research.

**Graphical abstract:**

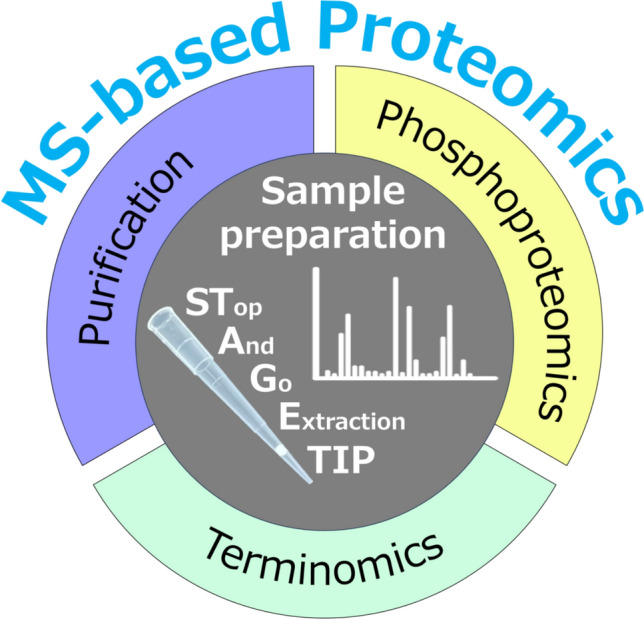

## Introduction

In proteomics, it is essential to have an analytical platform that performs large-scale measurement and data analysis to profile the proteome expression in biological samples [1–4]. Since the proteome directly reflects pathophysiological or biological processes, proteomics is expected to reveal potential disease biomarkers and therapeutic mechanisms [5–8]. Over the past two decades, nano-scale liquid chromatography/tandem mass spectrometry (nanoLC/MS/MS) has emerged as an excellent alternative to traditional two-dimensional gel electrophoresis [9, 10] and antibody-based approaches such as western blotting [11]. Because nanoLC/MS/MS-based proteomics can analyze over 10,000 peptides and proteins in a short period of time, it achieves significantly higher throughput with greater sensitivity [12–16]. Moreover, automation of sample pretreatment steps can further boost the throughput, accuracy, and precision [17–23]. The two most commonly used approaches are top-down and bottom-up proteomics [24, 25]. Both aim to identify and quantify complicated proteoforms, including post-translational modifications (PTMs), single amino acid substitution, and alternative splicing [26–28]. In top-down proteomics, intact proteins are directly separated and analyzed using LC/MS/MS [29–31]. In contrast, in bottom-up proteomics, proteins are treated with protease and the resulting peptides are analyzed by nanoLC/MS/MS for identification [32–35]. Because peptides are generally easier to isolate and characterize than intact proteins, bottom-up proteomics is more widely used. It is worth noting that top-down proteomics can analyze the linkage of two or more PTMs, which is not possible in bottom-up proteomics unless they are on the same peptide.

A typical nanoLC/MS/MS-based proteomic workflow includes not only the two steps mentioned above, i.e., protein/peptide separation and MS/MS measurement, as well as data analysis, but also a third major step, sample pretreatment [31–33, 36, 37]. Technologies required for this step have advanced significantly over the past 20 years, but it is important to note that each approach has its own unique characteristics and limitations, and as a result, these can have a significant impact on the quality of the data [38, 39]. In particular, any errors or biases introduced at the earliest stage of sample pretreatment can spread throughout the entire procedure, so unbiased, robust, and consistent sample preparation is crucial to the success of bottom-up proteomics.

StageTips (Stop And Go Extraction Tips) have gained prominence as a low-cost and user-friendly tool for purifying and enriching peptides during sample pretreatment [40–42]. It was first conceived as a microscale solid-phase extraction (SPE) system, in which reversed-phase (RP) material is packed at the tip of a pipette, followed by centrifugation or vacuum to load, wash, and elute peptides. This step, frequently termed “desalting,” is primarily used to eliminate buffer salts from digested peptides in preparation for nanoLC/MS/MS [43]. Compared to commercially available mini-SPE tips (e.g., ZipTip) that use pipetting to repeatedly aspirate and dispense, StageTips minimize sample loss and also have a filtering effect on insoluble substances. Additionally, the disposable and cost-effective design helps prevent contaminant carryover into nanoLC/MS/MS. Researches published in the early 2000s demonstrated that StageTip-based methods achieve high sensitivity and robust reproducibility [40–42], and they have since been adopted as a routine step in numerous proteomics laboratories. In recent years, StageTips have expanded beyond basic desalting applications, finding use in fractionation [44–52] and in-tip reaction protocols [53–56] for deep and highly sensitive proteomics. These extensions support diverse new applications such as high-throughput proteome imaging and ultra-sensitive profiling at the single-cell level [47, 53, 57–62]. Here, we outline the principles and background of StageTip, review current developments and enhancements, and discuss its role and future potential in mass spectrometry-driven proteomics.

### Varieties of StageTip materials

**Fig. 1:**
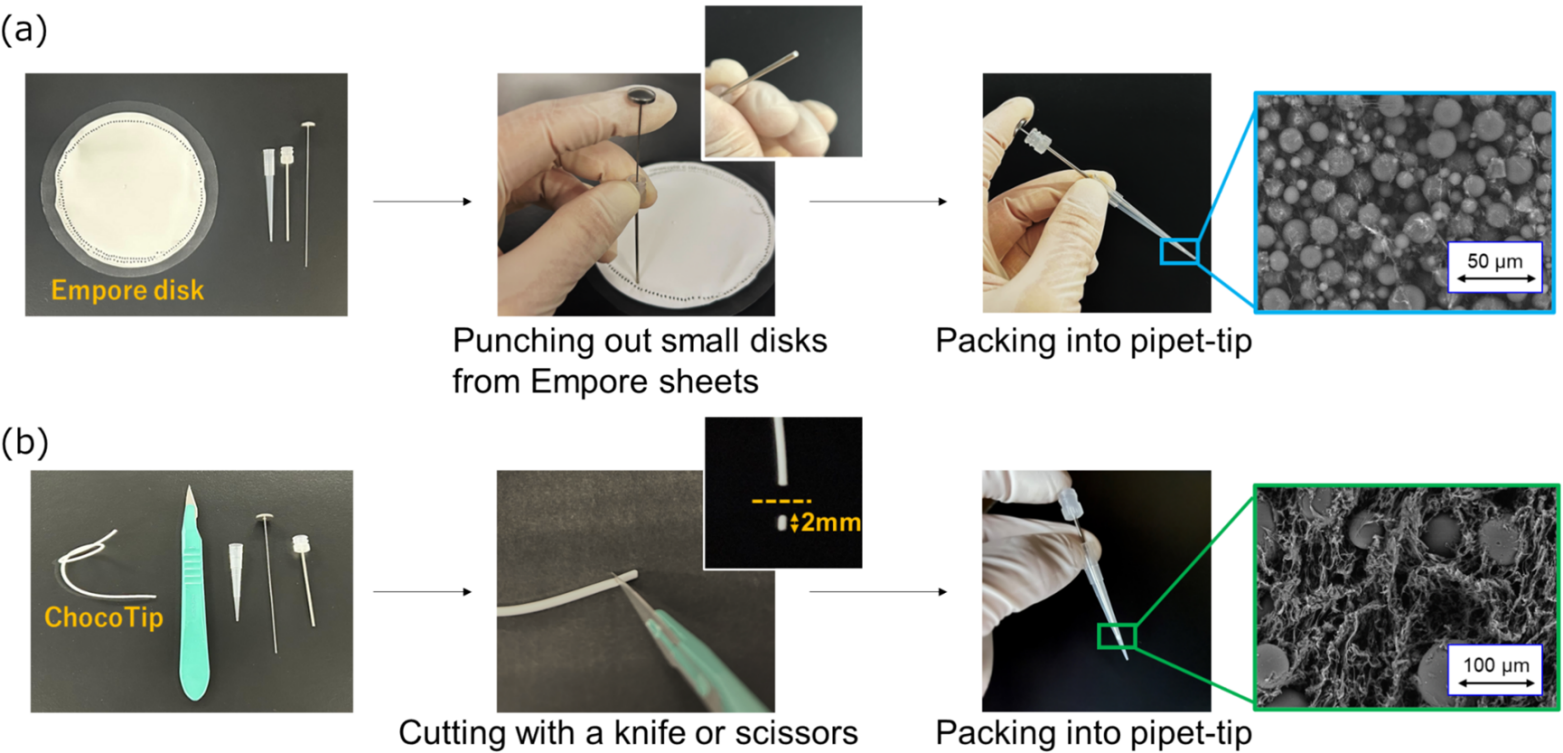
**a** Fabrication workflow of **a** StageTip and **b** ChocoTip. StageTip was prepared by punching out small disks from commercially available Empore™ sheets using a stainless steel needle, while ChocoTip was prepared by cutting a string-shaped material into 2.0 mm lengths with a scalpel or scissors. Each material was then lightly pressed into the pipette tip with a stainless steel needle until it caught on the inner wall, preventing further insertion. Figure modified from *Anal. Chem.*, **2024**, *96*, 20,390. [67] Licensed under CC BY 4.0

Standard StageTips are typically fabricated by placing a small disk of Empore™ (originally from 3 M, now supplied by CDS analytical (Oxford, PA, USA)) material at the tip of a standard pipette tip [40, 41]. Empore disks consist of chromatographic beads immobilized on a Teflon™ mesh, which can be punched out from large commercial sheets using a blunt-ended needle (Fig. 1a). The diameter of the disk depends on the needle’s inner bore, allowing users to customize the disk size as needed. Thanks to these simple steps, any laboratory can prepare StageTips tailored to a desired capacity and functionality.

From our experience, one of the main advantages of using StageTip is that it considerably enhances the robustness of the proteomics workflow. Merely washing peptides through a StageTip efficiently removes impurities such as cell debris and aggregates, thereby preventing clogging in the LC column. This, in turn, improves the overall stability of the LC/MS/MS system. Empore disks can incorporate various chromatographic media, including silica-based C18, polymeric styrene–divinylbenzene (St-DVB), and strong cation/anion exchangers (SCX/SAX) [49, 63–65].

Among RP materials, silica-based C18 has been used most extensively. It forms strong hydrophobic interactions with trypsin-digested peptide mixtures, retaining them effectively while enabling the easy washing out of salts and other water-soluble impurities. In addition, peptides can be eluted with organic solvents (mainly acetonitrile), which is now a well-established procedure in proteomics experiments. Polymer-based St-DVB has also been employed as an RP material, and there are reports suggesting that it captures a broader range of moderately polar peptides compared to silica-based C18, thereby improving the comprehensiveness of peptide profiles. [66]

**Fig. 2:**
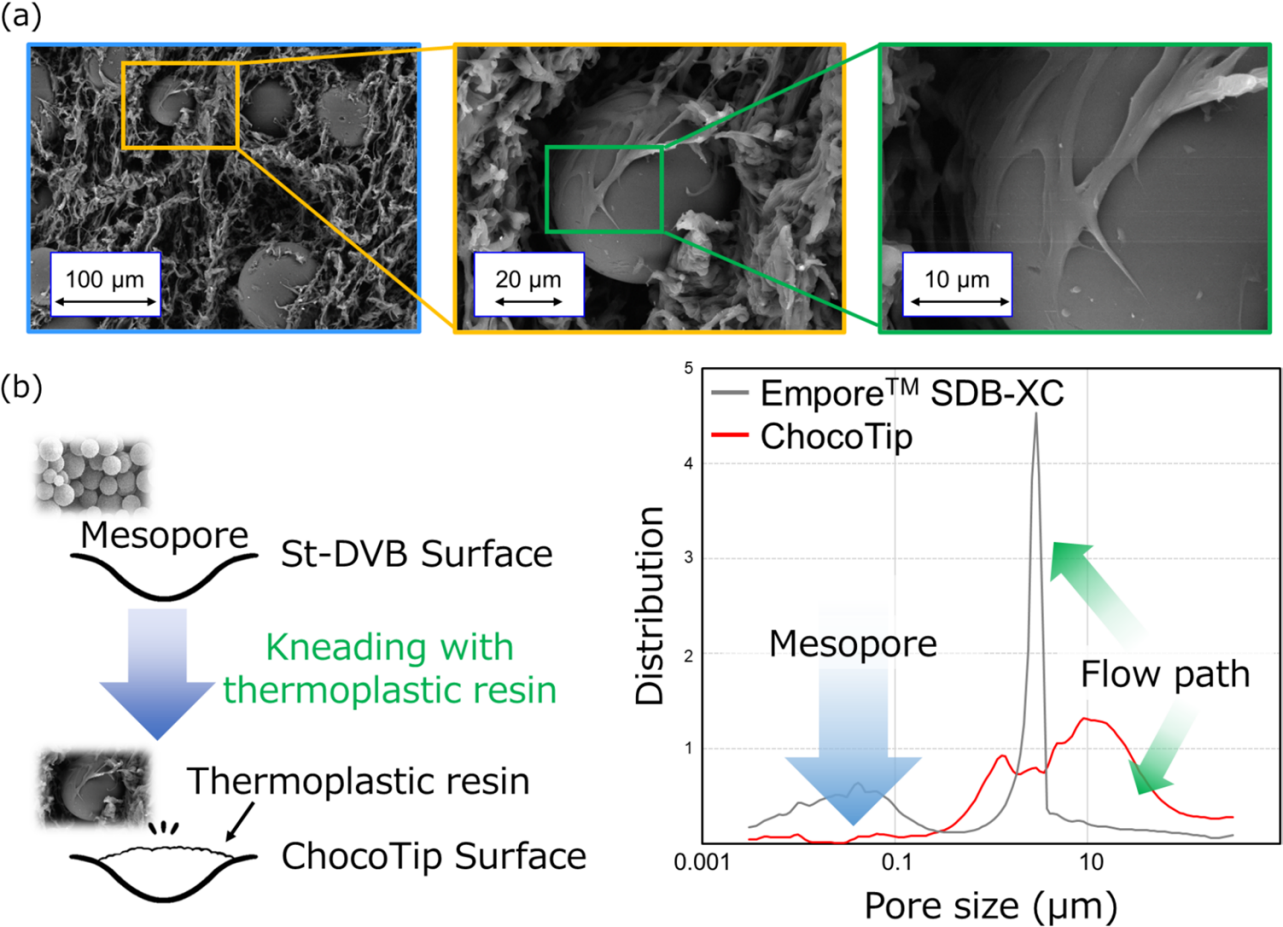
Morphological characterization of ChocoTip. **a** Scanning electron micrograph (SEM) revealing the surface of a ChocoTip, where the fibrous thermoplastic partially envelops the St-DVB particles. **b** Pore size distribution of ChocoTip. The thermoplastic resin creates micrometer-scale flow channels and seals the mesopores on the surfaces of the St-DVB particles, thereby preventing irreversible peptide adsorption within the StageTip. Figure modified from *Anal. Chem.*, **2024**, *96,* 20,390. [67] Licensed under CC BY 4.0

Recently, we developed a new StageTip called ChocoTip (CHrOmatographic particles COated by a Thermoplastic polymer Immobilized in Pipette Tip), specifically designed for purifying very small amounts of peptide samples (Fig. 1b, Fig. 2) [67]. ChocoTip uses a proprietary packing material that combines a thermoplastic resin and hydrophobic particles in a hybrid structure, thereby minimizing peptide irreversible adsorption in the column and reducing sample loss significantly. In experiments using 20 ng of trypsin-digested peptides derived from HeLa cell lysates, ChocoTip exhibited more than a twofold increase in sensitivity and peptide identifications compared to St-DVB-based StageTips. Furthermore, scanning electron microscopy and mercury intrusion porosimetry revealed that the thermoplastic resin coats the mesopores of the hydrophobic particles, preventing irreversible peptide binding inside those mesopores. This mechanism appears to account for the substantial decrease in sample loss (Fig. 2b). As another approach to enhance peptide recovery, we introduced the “CoolTip,” in which the entire StageTip is operated under low-temperature conditions during the desalting step [66]. Under cooler conditions, intermolecular interactions become stronger, increasing peptide retention on the RP material in the StageTip. This has improved the recovery of hydrophilic peptides in particular.

In ion-exchange chromatography, SCX and SAX modes are commonly used, where retention is controlled by the charge state of the peptide and the ionic strength of the elution buffer. In particular, SCX-StageTip fractionation shows high orthogonality to RPLC/MS/MS analysis as high-pH RP fractionation, leading to a marked reduction in sample complexity [49, 68]. In SCX-StageTip, peptides with positive charges are bound and then eluted into multiple fractions by stepwise increases in salt concentration or changes in pH. In our early work, we performed multidimensional fractionation using a StageTip layered with SCX–C18–SCX, enabling large-scale identification of over 9,500 proteins [42]. We also established an acid gradient elution protocol for SCX-StageTips. The peptide separation using SCX-StageTip was greatly improved by the acid-based elution method compared with a standard salt-based elution method, successfully identifying more than 20,000 phosphopeptides [44].

In recent years, there have been many reports of StageTips using separation modes other than reversed-phase or ion exchange, such as hydrophilic interaction chromatography (HILIC) and porous graphite carbon (PGC). HILIC readily retains the polar regions of peptides and is sometimes used for glycosylated peptide analysis [50, 69]. Meanwhile, PGC is known to enhance the retention of polar peptides through strong hydrophobic and π interactions, but excessively strong binding can lead to losses of more hydrophobic peptides [70, 71]. Nevertheless, a key benefit of the StageTip technology lies in the flexibility to incorporate these diverse packing materials according to specific experimental goals. By further refining the materials’ design, there remains significant potential to improve the sensitivity and coverage of MS-based proteomic analyses.

### Phosphoproteomics

Phosphorylation is one of the most prevalent post-translational modifications in living systems, believed to be involved in nearly all cellular processes [72]. Indeed, almost all cellular proteins have at least one phosphorylation site, making it a formidable challenge to comprehensively understand these phosphorylation events on a proteome scale [73]. Bottom-up proteomics has been used for over a decade to study dynamic cellular signaling [74, 75], and phosphorylation proteomics has rapidly gained prominence as a growing research field. [76–79]

**Fig. 3:**
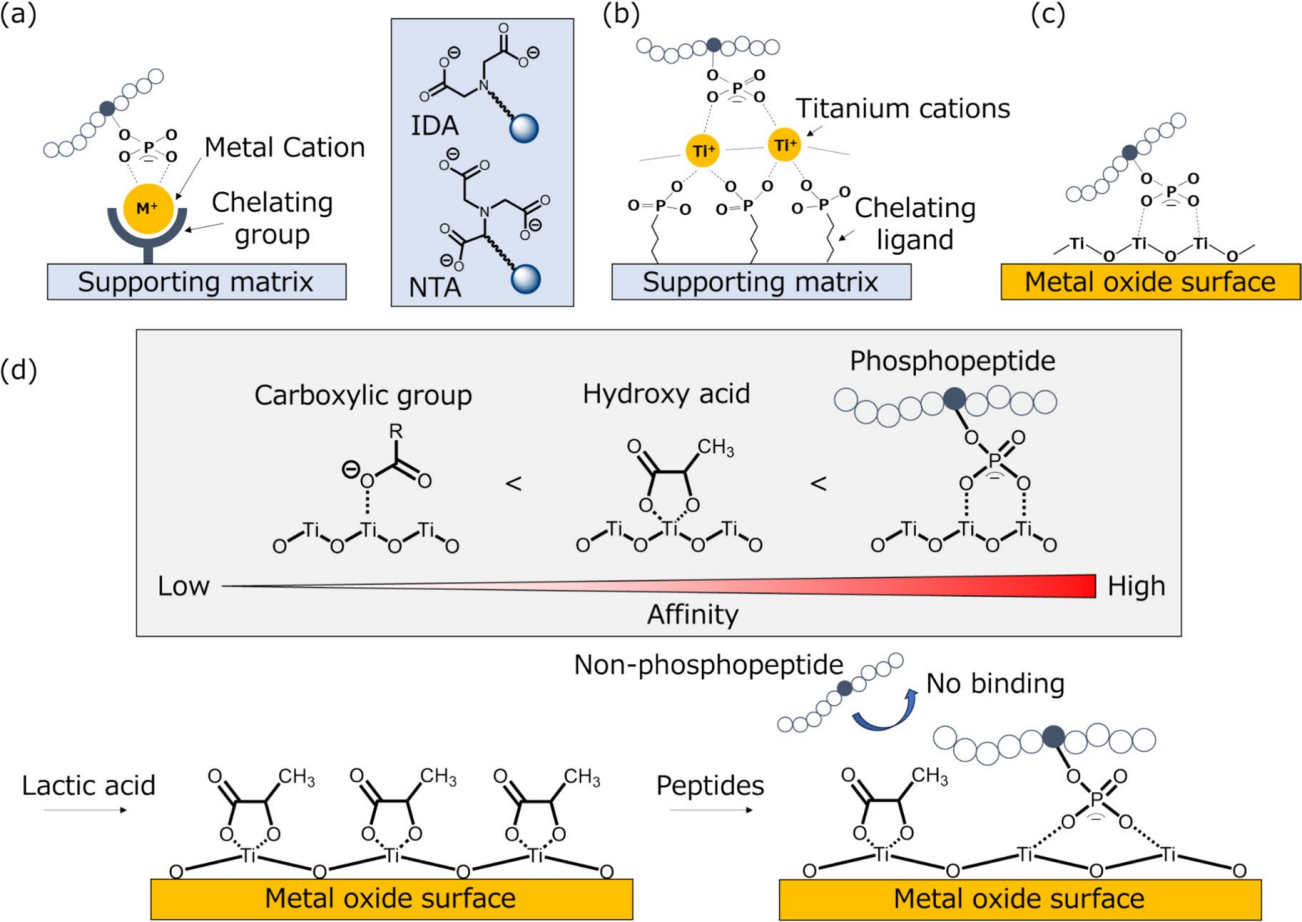
Schematic illustration of the IMAC and MOC method. **a** IMAC leverages the capacity of metal ions to bind phosphate groups via chelation and electrostatic interaction. In classical IMAC matrices, metal cations are noncovalently immobilized by chelating ligands such as iminodiacetic acid (IDA) and nitrilotriacetic acid (NTA). **b** The Ti^4+^-IMAC provides enhanced selectivity and capacity through the use of phosphate ligands for chelation. **c** Metal oxides represent another category of metal-based affinity materials, wherein metal chelation and electrostatic forces enable binding to phosphate groups. MOC typically uses pure metal oxide beads. **d** The schematic diagram of the HAMMOC method

Phosphorylation generally occurs at low stoichiometry, exhibits substantial complexity, and is highly dynamic, thus requiring the selective enrichment of peptides containing phosphorylated serine, threonine, and tyrosine residues prior to LC/MS/MS analysis. To achieve this, numerous protocols have been devised to isolate phosphopeptides from a complex pool that is predominantly composed of non-phosphorylated peptides. Most of these methods incorporate a step of peptide fractionation, either after or more frequently [80, 81], before a subsequent phosphopeptide enrichment process utilizing metal affinity chromatography such as immobilized metal ion affinity chromatography (IMAC, Fig. 3ab) [75, 82–85] and metal oxide chromatography (MOC, Fig. 3c) [86–88], typically performed with TiO_2_.

We reported hydroxy acid-modified metal oxide chromatography (HAMMOC) method (Fig. 3d), which pre-adsorbing an aliphatic hydroxy acid, such as lactic acid, onto the metal oxide in the StageTip and also adding hydroxy acid to the sample solution markedly enhanced the selectivity for phosphopeptide enrichment [87]. This was presumed to arise because the bond between hydroxy acid and metal oxide is stronger than that between carboxylate groups on acidic peptide residues and MOC, whereas the affinity between phosphate groups and MOC is stronger than that between hydroxy acid and metal oxide, thereby enabling selective removal of only acidic peptides. Furthermore, calcining TiO_2_ at 800 °C to convert the original anatase crystal form into the rutile form was found to confer sufficient phosphopeptide selectivity without the need to add hydroxy acids [89]. In addition, optimizing the elution conditions revealed that 5% ammonia, 5% piperidine, and 5% pyrrolidine each elute distinct subsets of phosphopeptides. Capitalizing on this property, three different elution solutions were sequentially applied to a single StageTip, successfully enriching and fractionating phosphopeptides and ultimately achieving approximately twice as many identifications compared with conventional methods. [90]

### Protein terminomics

Even a single gene can yield proteins with different sequences or modifications through diverse biological mechanisms, such as alternative splicing, mutations, translational regulation, proteolysis, or modifications during and after translation. Collectively, these varied isoforms are referred to as proteoforms [26, 27, 91], each typically exhibiting distinct functions, localizations, and stabilities [92]. Moreover, differences in translation initiation sites, translation termination sites or proteolytic processing can give rise to molecules bearing unique N, C-terminal sequences. In addition, the presence of signal peptides or transit peptides can influence both localization and stability [93]. Consequently, to understand the functional diversity of proteoforms, a comprehensive method to analyze protein termini, termed protein terminomics, is essential.

In protein terminomics, protein terminal peptides are separated from other digestion-derived peptides, followed by bottom-up proteomics. Numerous approaches have been developed to isolate N-terminal or C-terminal peptides of proteins. Among them, COFRADIC (combined fractional diagonal chromatography) [94] and TAILS (terminal amine isotopic labeling of substrates) [95] are widely recognized for N-terminal peptide isolation, and several additional techniques have also been reported [96]. However, each of these methods requires intricate, time-consuming protocols involving chemical modifications to block amine groups, and challenges remain regarding the efficiency and specificity of these modifications.

**Fig. 4:**
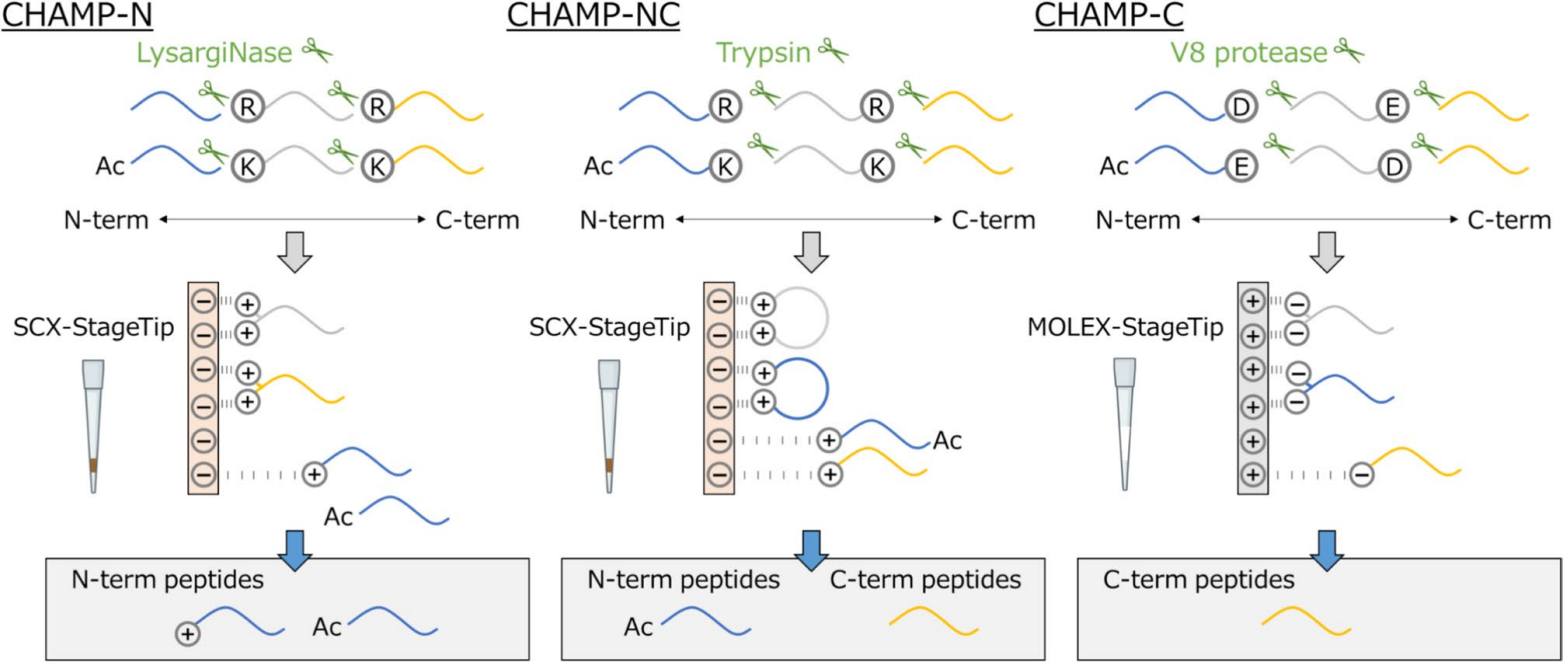
Schematic illustration of protein N- and C-terminal peptide enrichment with the CHAMP method. In the CHAMP-N method, digestion with lysargiNase produces peptides bearing Lys or Arg at their N-terminus, each carrying at least a + 2 charge. In contrast, peptides originating from the protein’s actual N-terminus typically have a + 1 charge because they lack Lys or Arg, except when a peptide contains histidine with an unmodified N-terminus, in which case it can have a + 2 charge. In the CHAMP-NC method, trypsin digestion yields internal peptides with at least a + 2 charge, whereas protein C-terminal peptides lacking histidine carry a + 1 charge and those containing histidine carry a + 2 charge. Taking these charge patterns into account, protein N-acetylated terminal peptides and protein C-terminal peptides can be selectively isolated from internal peptides using SCX under low-pH conditions. In the CHAMP-C method, digestion with V8 protease, which cleaves on the C-terminal side of aspartic and glutamic acids, generates protein C-terminal peptides with only one carboxy group, whereas internal peptides gain two carboxy groups at their C-termini. In MOLEX chromatography, protein C-terminal peptides are separated from internal peptides based on the latter’s ability to form a stable chelate with metal atoms via their dicarboxylate groups

We recently established simpler approaches to isolate protein N- and C-terminal peptides by combining enzymatic digestion with straightforward chromatographic separation. We refer to these methods as the “CHromatographic AMplification of Protein terminal peptides (CHAMP, Fig. 4)” methods for N-terminal peptides (CHAMP-N) [97, 98] and C-terminal peptides (CHAMP-C) [99]. Unlike conventional procedures, which involve complex steps, our methods enable the highly selective isolation of terminal peptides in a single step. Specifically, CHAMP-C uses a StageTip packed with CeO₂ particles to isolate C-terminal peptides from V8 protease digests, whereas CHAMP-N employs an SCX-StageTip to capture N-terminal peptides from LysargiNase digests. The CHAMP methods leverage StageTips to provide a straightforward, highly sensitive, reproducible, high-throughput and in-parallel approach to terminomics profiling, all without compromising the robust identification depth and selectivity typically achieved by HPLC-based fractionation methods.

### Comparison and overview of StageTips

We compiled a summary of the various StageTips introduced in this review, highlighting their key features, applications, and relevant references. Table 1 provides a convenient overview of the different materials, fractionation mechanisms, and specialized protocols that can be adapted to diverse proteomics workflows.

**Table 1:** Summary of StageTips discussed in this review

	Materials	Features	Main applications
Standard StageTip [40, 41, 42, 49, 63–65]	Empore disks (e.g., C18, St-DVB, SCX, SAX)	Simple to prepare by punching small disks in Empore™ sheets	Peptide purification Fractionation for large-scale proteomics
ChocoTip [67]	Spongy-like polymer	Hybrid material of thermoplastic resin and hydrophobic particles Suppressing irreversible peptide adsorption	Highly sensitive proteomics
HILIC-StageTip [50, 69]	HILIC materials	Strong retention of polarpeptides/glycopeptides	Glycopeptides and other polar peptide analyses
PGC-StageTip [70, 71]	Porous graphite carbon	Strong hydrophobic and π interactions Enhances retention of moderately polar peptides	Glycopeptides and other polar peptide analyses
IMAC/MOC- StageTip [44, 75, 82–90]	Metal ion chelate ligands, metal oxides	Selective enrichment of phosphopeptides	Phosphoproteomics
CHAMP-N/CHAMP-C [97, 98, 99]	SCX (CHAMP-N) or CeO₂ (CHAMP-C)	Enrichment of terminal peptides	Terminomics Deep proteoform analysis

Each StageTip offers distinct advantages, from enhanced sample recovery to specialized fractionation, and can be chosen according to the requirements of specific experiments

### Recent advances in StageTip-based approaches

Among the latest developments in this field are in-StageTip [53, 54] and on-microSPE methods [55, 56], both of which utilize a StageTip as a solid-phase reactor throughout the entire sample preparation workflow, from cell lysis to peptide purification. These strategies effectively minimize contamination and sample loss throughout the process. Another noteworthy innovation is the Evosep One system [100, 101], which is specifically designed for high‐throughput and robust LC/MS/MS‐based proteomics. Integrating the StageTips upstream as an injection device streamlines sample loading and desalting processes, thereby reducing manual handling and potential sample loss. The Evosep One achieves its high throughput through a series of pre-formed gradients and rapid column equilibration, minimizing instrument downtime and allowing large numbers of samples to be processed consecutively with minimal carryover. Such techniques lay the groundwork for next-generation technologies, including clinical proteomics, single-cell proteomics and biopsy-based proteome imaging, and, when combined with genomic or transcriptomic data, provide higher-resolution molecular insights into biology [47, 53, 57–62].

## Conclusions

StageTips have firmly established themselves as a pivotal tool for sample preparation in MS-based proteomics. They offer a user-friendly, low-cost platform for peptide desalting, fractionation, and on-tip reactions without sacrificing sensitivity, reproducibility, or throughput. Innovations in packing materials, ion-exchange fractionation, and specialized workflows for phosphoproteomics and terminomics underscore the versatility of StageTips for deep proteomic and proteoform analyses. Ongoing efforts to integrate StageTips into automated, high-throughput systems, such as Evosep One, further expand their impact, particularly in single-cell and clinical applications. Through continued optimization of materials and designs, StageTip-based methods will drive more comprehensive and sensitive proteome coverage, aiding in the convergence of multi-omics approaches and advancing our understanding of complex biological systems.

## Data Availability

The authors confirm that the data supporting the findings of this study are available within the referenced articles.
